# An AI-Based Integrated Multi-Sensor System with Edge Computing for the Adaptive Management of Human–Wildlife Conflict

**DOI:** 10.3390/s25206415

**Published:** 2025-10-17

**Authors:** Mirosław Hajder, Janusz Kolbusz, Mateusz Liput

**Affiliations:** Department of Intelligent Systems and Networks, University of Information Technology and Management, 35-225 Rzeszów, Poland; mhajder@wsiz.edu.pl (M.H.); jkolbusz@wsiz.edu.pl (J.K.)

**Keywords:** Human–Wildlife Conflict (HWC), edge computing, AI, non-lethal deterrence, sensor node, NVIDIA Jetson, wolf, bear, olfactory deterrent

## Abstract

Escalating Human–Wildlife Conflict (HWC), particularly involving protected large carnivores such as the wolf, poses a significant challenge in Europe. This problem, exacerbated by ecological pressure, necessitates the development of innovative, non-lethal, and effective prevention methods that overcome the limitations of current passive solutions, such as habituation. This article presents the design and implementation of a prototype for an autonomous, multi-sensory preventive system. Its three-layer architecture is based on a decentralized network of sensory-deterrent nodes that utilize Edge AI for real-time species detection and adaptive selection of deterrent stimuli. During field validation, the prototype’s biological efficacy as a proof-of-concept was confirmed in a crop protection scenario against the European roe deer (*Capreolus capreolus*). The system’s deployment led to a near-total elimination of damages. The paper also presents key technical performance metrics (e.g., response time, energy consumption) and the accuracy of the implemented AI detection model, verified using both field and historical data. The positive test results demonstrate that the developed platform provides an effective and flexible foundation for preventive systems. Its successful validation on a common herbivore species represents a crucial, measurable step toward the target implementation and further research on the system’s effectiveness in providing protection against large carnivores.

## 1. Introduction

### 1.1. The Global and European Context of Human–Wildlife Conflict

Human–Wildlife Conflict (HWC) is one of the most complex and dynamically growing environmental challenges of the 21st century, affecting communities on every continent. Paradoxically, its particular escalation in Europe is partly a result of the success of long-term nature conservation strategies, such as the European Union’s Habitats Directive. These actions have led to the spectacular comeback of many species, including large carnivores like the wolf (*Canis lupus*) and the brown bear (*Ursus arctos*), to areas from which they were driven out centuries ago. However, this conservation success collides with the concurrent and unprecedented expansion of human activity, leading to conflict escalation. This problem is bidirectional as it encompasses both livestock damage caused by predators and crop losses generated by numerous populations of herbivorous animals, including roe deer, deer, and moose.

The primary cause of the intensification of this conflict is the shrinking and fragmentation of natural wildlife habitats. Constant urbanization, the development of a dense transport infrastructure network, and the intensification of agriculture are blurring the boundaries between the natural world and the human-dominated landscape. This process, exacerbated by global climate change and progressive environmental degradation, forces wild animals to seek new territories and resources, inevitably leading to direct competition with humans for space, water, and food.

In this context, HWC also takes on a social and political dimension. A lack of public ecological awareness and insufficient education on the functioning of ecosystems, often observed at the decision-making level, leads to the adoption of ad hoc measures instead of implementing integrated and long-term management strategies. The overexploitation of natural resources further deepens the problem by disrupting the natural balance and forcing wildlife to adapt in ways that generate increasingly frequent and severe flashpoints at the interface of both worlds. Understanding these fundamental, multidimensional causes is key to developing effective and ethical solutions that will enable sustainable coexistence in the future.

### 1.2. Podkarpackie Voivodeship as a European Conflict Hotspot

A perfect example illustrating the scale and dynamics of the described phenomenon is the Podkarpackie Voivodeship in southeastern Poland. This region, characterized by high forest cover and serving as a stronghold for the country’s largest populations of wolves and bears, has become a veritable Human–Wildlife Conflict “hotspot” on a European Union scale over the last decade [1,2,3,4]. The convergence in this area of growing predator pressure; a specific, fragmented agricultural structure; and an increasing ecological imbalance makes it an ideal case study for an in-depth analysis of the causes, effects, and potential technological solutions to this escalating problem. A detailed, multidimensional analysis of this region will be presented in the following section.

### 1.3. Review of Existing Non-Lethal Methods and Their Limitations

The search for effective conflict management methods is driven not only by the legal protection status of species like the wolf and bear but also by a growing ethical awareness that aims to minimize animal suffering and treat them as full-fledged members of the ecosystem. In response to these needs, a range of non-lethal methods have been developed, which can be divided into three main categories: physical barriers (e.g., specialized fencing), biological repellents (e.g., the use of livestock guardian dogs), and chemical (olfactory repellents) repellents [5,6]. Although each of these methods has a certain effectiveness under specific conditions, chemical repellents are the subject of wide interest due to their potentially low cost and ease of application. Their mechanism of action is based on the assumption that a strong olfactory stimulus, imitating the presence of a human or another natural threat, can induce an instinctive avoidance reaction in predators. Commercial products implementing this strategy are available on the market, such as Hukinol, which is based on concentrated human sweat odor [7,8].

A common and fundamental limitation of all of the above methods is their passive, non-reactive nature. Their operation is independent of dynamically changing conditions, such as wind direction, the number of animals, or their specific behavior. Moreover, these systems lack the ability to perform species recognition and therefore cannot react selectively to a specific threat or choose stimuli specific to a particular animal. This inability to adapt is the main cause of habituation, a phenomenon widely documented in research [9,10,11]. Habituation consists of an animal’s gradual acclimatization to a constant, repetitive stimulus that is not associated with any real negative consequences. As a result, the animal learns to ignore a signal that initially provoked a fear response. Field observations fully confirm these findings; long-term, passive exposure of local fauna to preparations like Hukinol or to auditory or visual signals leads to an initial effectiveness of a few days, followed by a sharp decline and, eventually, a disappearance of the aversive reaction. Crucially, in passive systems, the moment effectiveness is lost is impossible to monitor in real time; information about the deterrent’s failure only emerges post factum, when another instance of damage occurs. This key flaw in existing solutions is the main starting point for the search for new, dynamic deterrence strategies. It points to the necessity of finding solutions based on the integration of different deterrence methods, their dynamic variability over time, and the real-time monitoring of their effectiveness [12,13].

In an approach similar to ours, reference [14] presented an IoT system architecture for classifying images from camera traps on an edge device, with data transfer over a LoRaWAN network. In their research, the authors conducted a detailed comparison of different models and hardware platforms (including the NVIDIA Jetson Nano), demonstrating that optimizing a model for edge devices can lead to a significant drop in classification accuracy. Our system expands upon this concept by utilizing a newer generation of both the hardware platform (the Jetson Orin family) and AI models (YOLOv8), which aims to maintain high precision even after deployment on the end device. Furthermore, our three-layer architecture with an aggregator node has been optimized for reliability under conditions of unstable connectivity, addressing the challenges of data transfer in difficult terrain.

In turn, reference [15] focused on the problem of energy consumption optimization, which is critical for field systems. The authors proposed and evaluated a series of methods aimed at minimizing power draw, such as adaptive regulation of sensor sensitivity and the use of simple motion detection algorithms to filter out false alarms before initiating energy-intensive neural network inference. Although our solution also implements advanced power management strategies, our main contribution lies in a different area. In contrast to systems focused solely on detection and classification, our work concentrates on closing the operational loop by adding a layer of intelligent, active deterrence. This constitutes a key step toward building fully autonomous preventive systems, not just monitoring ones.

Finally, reference [16] highlighted the fundamental importance of proper calibration of the sensory devices themselves. The authors proposed a methodology for optimizing camera trap settings to maximize the detection probability of specific species. This underscores that even the most advanced AI algorithms are useless if the quality of the input data is low. In our system, we also place great emphasis on this aspect, but we go a step further. Our architecture, thanks to a central MLOps platform, allows not only for initial calibration but also for continuous, remote adaptation and re-training of AI models in response to data collected in real time, which is a key element in ensuring long-term effectiveness in dynamically changing conditions.

### 1.4. Aim and Scope of the Article

In response to the limitations of passive systems diagnosed in the previous subsection, this article proposes a new solution: an autonomous sensory system that addresses the problem of habituation through a strategy of active, event-driven deterrence. The concept is based on three fundamental principles: being event-driven, meaning activation occurs only in response to detecting an animal; selectivity, which is the ability to distinguish between species and react only to a defined threat; and adaptability, which is the capacity to dynamically change the applied stimuli to prevent acclimatization. This operational model is the direct opposite of passive methods with a constant, non-reactive nature.

The technological heart of the proposed system is Artificial Intelligence operating on an edge device (Edge AI) [17]. The choice of this technology is dictated by the need to ensure full autonomy in field conditions, which eliminates the requirement for constant network connectivity and guarantees a minimal system response time (defined in this work as the time from motion detection to the activation of the first deterrent stimulus), which are widely described as key advantages of this architecture [18,19,20]. The conceptual architecture of the device consists of three integrated modules: a sensory module (responsible for acquiring data from the environment), a processing module (performing species detection and classification locally on the device), and an effector module (activating deterrent stimuli appropriate to the situation).

It should be emphasized that the choice of a three-layer hierarchical architecture was a deliberate design decision, which was made after analyzing the limitations of two alternative, seemingly simpler two-layer models. The first alternative, based on direct communication from a dense network of sensor nodes to a central cloud server, was rejected due to critical issues with reliability and cost. Field experience in the Carpathian region showed that cellular connectivity is unstable and often completely unavailable during nighttime hours, which would render the system inoperable during the key period of predator activity. The second alternative, which involved completely eliminating the central node and offloading all tasks to aggregator nodes, also proved to be inefficient. Such a model prevents global data analysis, large scale action coordination, and, most importantly, the implementation of a central MLOps platform for continuous training and distribution of improved AI models. The three-layer architecture with Edge AI proposed in this paper is a direct response to these limitations, combining the advantages of local, fast response at the network edge with the capabilities of global analysis and strategic management, thereby ensuring the autonomy, scalability, and long-term adaptability of the entire solution.

The detailed design, construction, and methodology for evaluating the prototype’s effectiveness will be thoroughly presented in the subsequent sections of this paper. This article is organized as follows. Section 2 presents a detailed case study of the human–wolf conflict in the Podkarpackie Voivodeship. Section 3 describes the conceptual, three-layer architecture of the proposed system. Details regarding the implementation of individual components and the methodology for technical validation and field tests are contained in Section 4. Section 5 includes the discussion, which presents the interpretation of the results, an economic analysis, a discussion of the study’s limitations, and directions for future work. The paper concludes with the Section 6.6.

## 2. Case Study: Podkarpackie Voivodeship

### 2.1. Introduction to the Study Area

**Figure 1:**
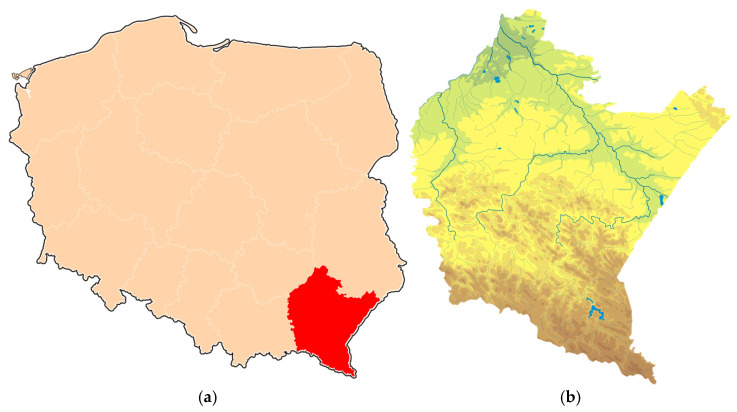
(**a**) Podkarpackie Voivodeship—location of the region on the map of Poland is marked in red. (**b**) Topography of the Podkarpackie Voivodeship.

The Podkarpackie Voivodeship, located in southeastern Poland (Figure 1a), covers an area of nearly 18,000 km^2^ and is a key region in the country in terms of biodiversity and forest cover. It is one of Poland’s most forested regions—forests cover over 38% of its area, and unlike in many other parts of the country, this indicator is systematically increasing. A significant part of its territory, particularly in the southern belt, is occupied by the mountainous and upland areas of the Carpathian Mountains (Figure 1b), which, due to their topography, are largely excluded from intensive agricultural use and form the core of the region’s forested areas. This area contains vast, cohesive forest complexes, including the Bieszczady National Park and the Magura National Park. They constitute one of Poland’s most important strongholds for large carnivores, including the country’s largest populations of wolves and bears. The region is also characterized by a relatively low population density and an agricultural structure based on small, fragmented farms, often with a livestock profile (mainly sheep and goats). This combination of proximity to vast, wild areas and extensive agricultural activity creates specific conditions conducive to the emergence of human–wildlife conflicts, making this region an ideal area for a case study.

### 2.2. Scale and Temporal Dynamics of the Conflict

**Figure 2:**
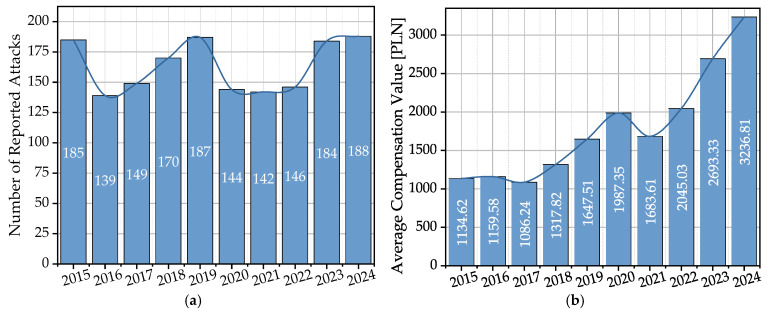
(**a**) Number of registered incidents of wolf attacks in the Podkarpackie Voivodeship between 2015 and 2024. The overlaid line is a Bézier curve added to visualize the general trend of fluctuations. (**b**) Change in the average compensation value [PLN] for a single incident between 2015 and 2024. The overlaid line is a Bézier curve added to visualize the general trend.

In the analyzed decade (2015–2024), the relevant regional governmental body for environmental protection (Regional Directorate for Environmental Protection in Rzeszów, hereinafter RDEP) registered a total of 1635 incidents resulting in compensation payments for damages caused by wolves. As shown in Figure 2a, the annual number of such events exhibits clear fluctuations, ranging from 139 (in 2016) to 188 (in 2024). Despite these fluctuations, the data unequivocally indicates that the conflict has persisted at a consistently high level throughout the decade, with no discernible downward trend. It should be emphasized that these figures represent only the officially registered fraction of all negative interactions. Many incidents, including successful deterrent actions or damages of minor value, are often not reported, suggesting that the true scale of the problem is likely much wider than official data indicate.

In addition to the constant high frequency, the data also point to the growing economic severity of individual incidents. An analysis of the average compensation value paid (Figure 2b) reveals a clear and consistent upward trend. This value increased from 1135 PLN in 2015 to 3237 PLN in 2024, representing a rise of nearly 200%. Such dynamics may suggest that individual attacks are becoming more severe, which is likely a result of the increasing scale of these events (a larger number of animals killed per attack by larger packs), and to a lesser extent, potential pressure on more valuable livestock species.

### 2.3. Spatial Concentration of the Conflict

An analysis of data from 2015–2024 unequivocally indicates that the human–wolf conflict in the Podkarpackie Voivodeship is not an evenly distributed phenomenon. On the contrary, it is characterized by a very high degree of spatial concentration, both at the county (powiat) and municipal (gmina) levels.

**Figure 3:**
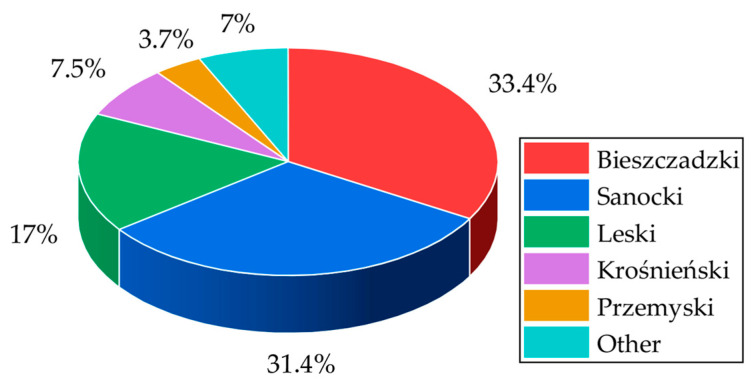
Visualization of the percentage share of individual counties in the total amount of damage caused by wolves in the 2015–2024 period.

As presented in Figure 3 and Table 1, as many as 89.3% of all registered incidents occurred within the territory of just four of the voivodeship’s nineteen counties. The absolute epicenters of the conflict are the Bieszczady (33.4% of all events) and Sanok (31.4%) counties, which together account for nearly two-thirds of all damages.

A more granular analysis, descending to a lower administrative level, reveals the phenomenon of strong spatial clustering. For instance, in Bieszczady County, which consists of three municipalities (gminas), damages in recent years have been concentrated almost exclusively within the Lutowiska municipality. A similar situation occurs in Sanok County, where the Komańcza municipality is the key area of intensified predator pressure. This shows that even within the counties most affected by the conflict, the problem is not dispersed but is clustered in specific “hotspots,” which is of fundamental importance for designing effective prevention methods.

In summary, this case study clearly indicates that the human–wolf conflict in the Podkarpackie Voivodeship is a persistent issue, strongly concentrated spatially within just a few municipalities. Furthermore, an analysis of the ecological balance based on data from Statistics Poland points to a growing disparity that is a key driver of the conflict. In the analyzed period of 2015–2023, the wolf population in the region nearly tripled. During the same time, the populations of its natural prey, such as roe deer and red deer, remained stable or slightly decreased. This dynamic strongly suggests that the growing carnivore population is exerting increasing pressure on a limited natural food base, which may reinforce the tendency for wolves to seek alternative, easily accessible food sources near human settlements. This diagnosis—of a problem that is simultaneously chronic and driven by ecological pressure—unequivocally justifies the need for the development and implementation of precise, locally acting preventive systems, which will be the subject of the subsequent sections of this work.

## 3. System Concept and Architecture

### 3.1. Rationale for System Architecture

The concept of the presented system is based on a three-level hierarchical architecture. This choice was driven by five key, interconnected factors: four arising from practical field requirements (cost scalability, energy efficiency, communication reliability, and flexibility of deterrence strategies) and one stemming directly from ecological dynamics—the need to ensure system mobility in response to the natural movement of animal herds in search of food.

First, the effective protection of large, open areas such as pastures requires a distributed solution. Each autonomous node in our system has a limited, local operational range, both in terms of detection and the effectiveness of the emitted deterrent stimuli. Consequently, to ensure full and continuous coverage of the protected area, these nodes must be deployed at high density. This high-density deployment requirement makes minimizing the unit cost of each node a critical economic parameter. A hierarchical architecture directly addresses this challenge by allowing for a large number of simple, low-cost effector nodes (Level 1) supervised by a smaller number of more advanced units.

Second, operating in areas remote from permanent power infrastructure imposes the requirement of autonomy and high energy efficiency. Therefore, the most power-intensive tasks related to artificial intelligence, such as training and retraining classification models, are offloaded to the highest level of architecture (Level 3). This allows the end nodes (Level 1) to use power optimized Edge AI modules whose sole task is to perform inference (real-time image analysis using a pre-trained model) [21]. This division of tasks, combined with ultra-low-power microcontrollers managing the sleep state, guarantees a long operational time on battery power.

Third, deploying the system in mountainous terrain with limited or unstable connectivity to public networks poses a critical communication challenge. The three-level architecture solves this problem by introducing an aggregator node (Level 2) that serves as a local gateway. The end nodes communicate with it using reliable short-range protocols. Only the aggregator node (Level 2)—whose location is optimized to ensure both reliable communication with its subordinate Level 1 nodes and the best possible connectivity to the higher-level network maintains the costly and power-intensive connection to the central management node (Level 3) [22].

Finally, the hierarchical architecture enables an intelligent allocation of tasks to effectively counteract the phenomenon of habituation. At the end-node level (Level 1), thanks to the implemented Edge AI modules, autonomous, real-time tactical decisions are made, such as species recognition and the activation of an appropriate stimulus. In contrast, strategic tasks—such as training and distributing updated AI models, analyzing the behavior of new species, or changing the global deterrence strategy—are handled at the higher levels of the architecture. This flexibility, based on a continuous cycle of in field data collection and remote intelligence updates, allows the system to be dynamically adapted to protect against different animal species and their specific behaviors, which is a key premise of the presented solution.

**Figure 4:**
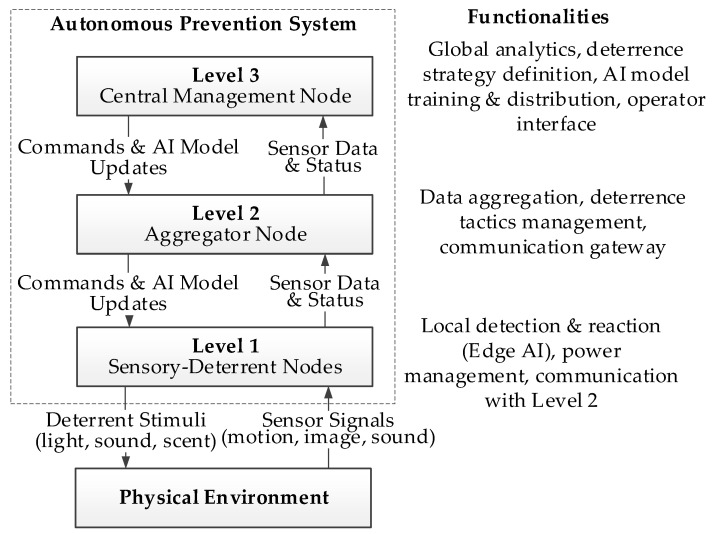
Conceptual diagram of the three-level hierarchical system architecture.

The aforementioned design principles led to the development of a three-level hierarchical system architecture, the conceptual diagram of which is shown in Figure 4.

### 3.2. Architecture of the Sensory-Deterrent Node

The sensory-deterrent node, which constitutes Level 1 in the system’s three-level architecture, is the most numerous and widely deployed element of the entire infrastructure, serving as an autonomous operational unit. Its key design principle is maximum energy efficiency to allow for long-term field operation on battery power. To this end, a cyclical operational model was implemented, in which the node remains in a deep sleep state for most of the time to minimize power consumption. In this mode, power is maintained only for the ultra-low-power Passive Infrared (PIR) sensors, which continuously monitor the surroundings. Only an interruption signal, generated by a sensor upon detecting motion, initiates the wake-up sequence. During this sequence, the more power-intensive subsystems—the vision system and the Edge AI module—are activated to perform real-time object analysis and classification. To ensure maximum energy efficiency, the node operates in three main modes (deep sleep, standby, and full activity). The system dynamically manages transitions between these states to optimize response time and battery consumption, especially in scenarios requiring extended observation after an interaction with predators. A detailed description of this strategy, along with time parameters for specific species, is presented in Section 4.4.

The hardware architecture of the sensory-deterrent node was designed as a trade-off between low unit cost, detection performance, and stringent power requirements. At its core is a two-part processing subsystem, consisting of a low-power microcontroller (MCU) that manages sleep and wake-up states, and a high-performance Edge AI module (e.g., from the NVIDIA Jetson family), which is activated only for the duration of inference.

The sensory subsystem of the Level 1 node consists of three complementary elements, selected to optimize detection and energy consumption. The role of the ultra-low-power, primary wake-up trigger is performed by a Passive Infrared (PIR) sensor, which constantly monitors the surroundings. The main sensor, responsible for acquiring data for species classification, is a vision module in the form of a dual (day/night) camera with an integrated infrared (IR) illuminator. This set is complemented by a sensitive microphone, whose task is not vocal identification but the detection of non-specific acoustic anomalies (e.g., the crack of breaking branches), which serve as an additional, medium-range wake-up signal.

**Figure 5:**
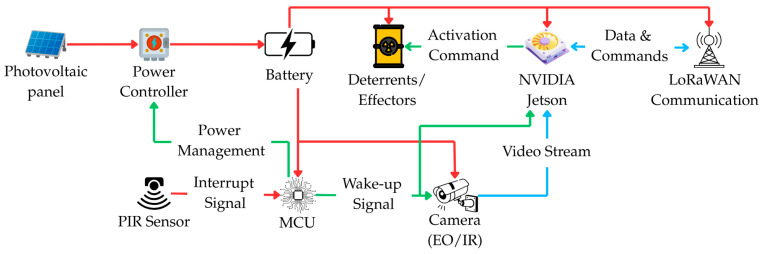
Connection organization scheme of the node’s hardware architecture.

**Figure 6:**
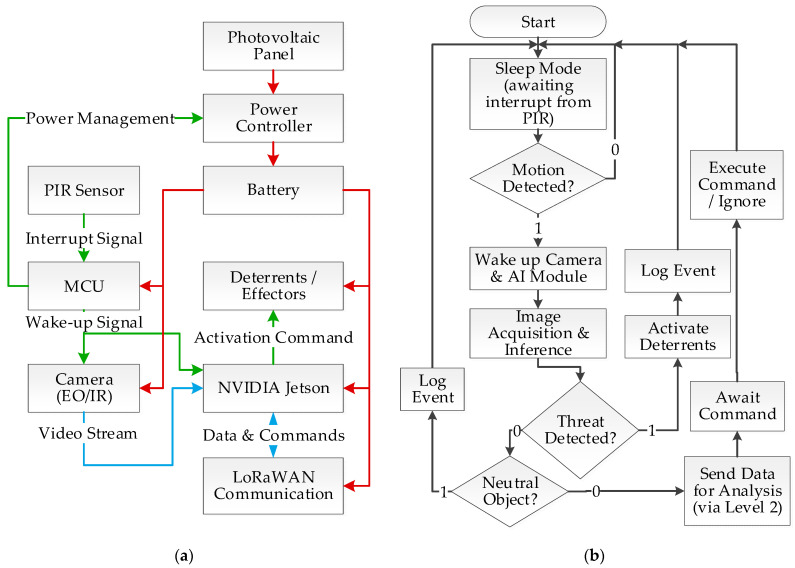
Architecture and operational logic of the Level 1 node: (**a**) block diagram of the hardware components, illustrating the flow of power (red), control (green), and data (blue) signals (MCU—Microcontroller Unit, PIR—Passive Infrared, EO/IR—Electro-Optical/Infra-Red); (**b**) simplified algorithm of the operational cycle. The flowchart represents the main system loop, which is executed continuously until the device is powered off.

Active prevention is carried out by an integrated effector subsystem equipped with a diverse array of stimuli: acoustic, visual (light emitter), and olfactory. Communication with the aggregator node (Level 2) is handled by a short-range radio module (e.g., LoRaWAN). The entire unit is powered by a dedicated power subsystem consisting of a photovoltaic panel, a charge controller, and a high-capacity battery, ensuring long-term autonomy in field conditions. A detailed block diagram and a connection organization scheme of the node’s hardware architecture, illustrating the flow of power, control, and data signals, are presented in Figure 5 and Figure 6.

The software architecture of the Level 1 node, closely coupled with its hardware structure, is twofold. The low-level microcontroller (MCU) runs lightweight embedded software (firmware), often based on a real-time operating system (RTOS), such as FreeRTOS. Its sole responsibility is to manage power, handle the passive sensors, and implement the sleep/wake-up cycle of the main processing module. In turn, the main system application runs on the Edge AI module (NVIDIA Jetson) in a Linux environment, leveraging advanced libraries such as Python 3.14, OpenCV 4.12 for image processing, and TensorFlow Lite for neural network inference. This application is responsible for implementing the system’s core logic: from image acquisition and object classification to making decisions about activating the appropriate effectors and managing communication with the aggregator node (Level 2).

At the application layer, the MQTT (Message Queuing Telemetry Transport) protocol, operating over the LoRaWAN physical layer, was used for communication between the sensory node and the aggregator. This choice was dictated by two key factors derived from our previous experience. Firstly, MQTT is an extremely “lightweight” protocol with minimal computational overhead, which is crucial for the resource-constrained Level 1 nodes. Secondly, its architecture, based on a “publish-subscribe” model, provides exceptional flexibility, allowing for the easy addition and relocation of nodes between different aggregator networks without the need for reconfiguration. This is an essential feature in the target scenario, where nodes must remain mobile, following herds moved as part of rotational grazing. A summary of information about communication protocols is presented in Table 2.

### 3.3. Architecture of the Aggregator Node

The aggregator node (Level 2) constitutes the intermediate, pivotal level in the system’s hierarchical architecture, acting as a local operational hub for its subordinate group of sensory-deterrent nodes (Level 1). Its primary objective is to offload the central system by pre-processing data and to ensure reliable communication in challenging field conditions. Depending on operational needs, this node has been implemented in two variants: a stationary version for the permanent protection of key areas, and a mobile version, as an autonomous unit installed on a dedicated trailer, enabling rapid system deployment in new or emerging hotspots.

The hardware architecture of the aggregator node is significantly more advanced than that of the end nodes. A high-performance module from the NVIDIA Jetson family was used as a powerful computing unit, capable of executing complex tasks such as data fusion from multiple sources or managing local deterrence tactics. In the stationary variant, at locations with access to the power grid, an industrial-grade computer with even greater computational power can also be used [23]. Its architecture is complemented by two specialized communication subsystems that define its dual role in the hierarchy. The first, utilizing short-range radio technology such as LoRaWAN, is responsible for collecting data from and managing the subordinate Level 1 nodes. The second, which forms the critical link to the higher-level system, is based on a long-range module (e.g., an LTE/4G/5G modem or a radio link) and is the only component in its network segment to maintain connectivity with the central management node (Level 3). The design is completed by an extensive power subsystem, which, depending on the location, relies on a photo-voltaic panel and a high-capacity battery, or, in the case of a limited grid connection (e.g., from a lighting network), on an oversized buffer system capable of storing enough energy for 24 h of operation.

The aggregator node’s software performs three main functions. First, it is responsible for aggregating and pre-filtering data from the end nodes, forwarding only key information and events to the central system. Second, it implements tactical logic, managing the deterrence strategy for its segment according to the latest directives received from Level 3. This is crucial for ensuring operational continuity if connectivity to the central node is lost; the aggregator node can then autonomously make tactical decisions and buffer incoming data from Level 1 until the connection is restored. Third, it serves as a Secure Gateway, managing the connection to the higher-level network. This includes resolving media access conflicts, aggregating data to optimize transmission, and handling group communications, for instance, when updating AI models. Furthermore, it protects the entire local subnetwork from unauthorized access.

Communication between the aggregator node and the central server (Level 3) was implemented using two complementary application protocols: MQTT and HTTPS. The choice between them is dynamically adapted to the type and urgency of the data. For transferring information requiring a near-real-time response, such as alerts about the detection of an unidentified object, the lightweight MQTT protocol was used. In contrast, for sending large, bulk data packets that were not sensitive to delays, such as daily archives of classified images, the HTTPS protocol was employed, which also ensures high security through its built-in SSL/TLS encryption.

Data security measures are based on three pillars: encryption, network separation, and authentication. Encryption is natively provided by the protocols used (AES-128 in LoRaWAN and SSL/TLS in HTTPS). Separation from the public internet is achieved by using a private APN within the LTE cellular network. Authentication in the system, designed as a closed network, is based on a Public Key Infrastructure (PKI) with self-signed certificates. The central server (Level 3) acts as the local Certificate Authority (CA). Each aggregator node (Level 2) receives a unique certificate from it, which is then used to authenticate and secure communication with its subordinate sensory nodes (Level 1), to which it provides the corresponding client certificates. This hierarchical trust structure ensures that communication is possible only between verified and authorized system components.

**Figure 7:**
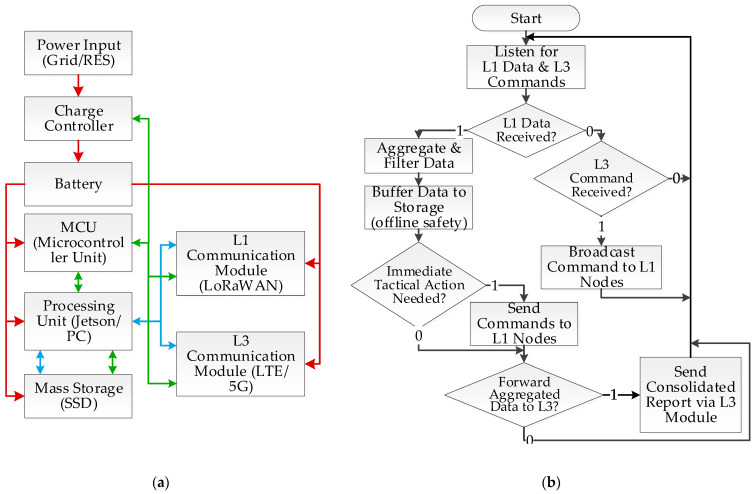
Architecture and operational logic of the aggregator node (Level 2): (**a**) block diagram of the hardware components, illustrating the flow of power (red), control (green), and data (blue) signals; (**b**) software logic flowchart.

The detailed hardware architecture and a flowchart of the aggregator node’s software logic are presented in Figure 7.

### 3.4. Architecture of the Central Management Node

The central management node (Level 3) represents the highest strategic level in the system’s hierarchy. It is implemented on a dedicated physical server as a server-side application, ensuring high computational power and reliability, although the system architecture also allows for its deployment in a secure cloud environment. Its role is multifaceted. First, it functions as a central data repository and a global analytics platform, enabling the identification of long-term trends and the evaluation of deterrence strategy effectiveness. Second, it acts as an on-demand, high-performance computing center, supporting lower-level nodes with power-intensive tasks, such as analyzing ambiguous sensor data in near-real-time.

Third, the central node operates as an “AI model factory.” Using the aggregated data, it performs the computationally demanding processes of training, validating, and re-training classification models. New, improved model versions are then remotely distributed to the nodes in the field, ensuring a continuous learning and adaptation process for the entire system.

**Figure 8:**
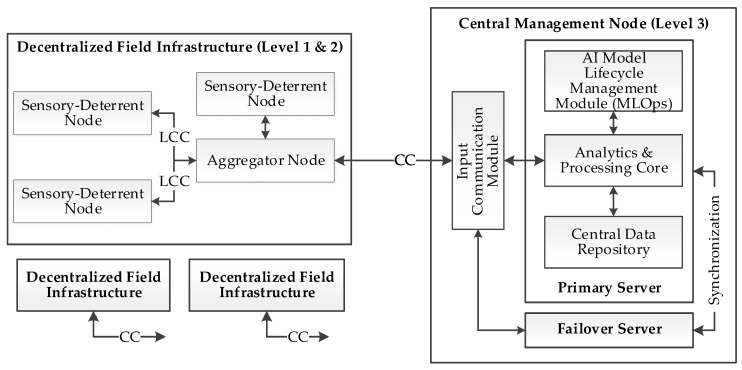
Functional architecture of the Central Management Node (Level 3) and its interaction with the decentralized field infrastructure. The diagram shows the high-availability design with a primary and a failover server. Acronyms: LCC—Local Communication Channel; CC—Communication Channel.

Finally, Level 3 provides a Graphical User Interface (GUI) for the system operator. This interface allows for data visualization on maps, configuration of global deterrence strategies, manual verification of ambiguous events, and monitoring the technical status and energy levels of individual nodes in the distributed network. The functional architecture of the central management node is presented in Figure 8.

The system’s designed and implemented architecture utilizes a single-input communication module. It’s important to note that while network connection redundancy is ensured at the operator infrastructure level, the communication module itself is not duplicated in hardware. This represents a conscious architectural compromise, relying on the inherent reliability of a high-quality, industrial-grade component. Such devices, characterized by high MTBF (Mean Time Between Failures), often owe their reliability to built-in redundancy of key components, eliminating the need for the additional complexity and cost associated with full hardware duplication of the module.

A dedicated set of Python scripts was used to manage the model lifecycle (MLOps). This choice, as opposed to implementing ready-made, extensive platforms (such as Kubeflow), allowed for the creation of a lightweight and fully controlled environment, ideally suited to the specifics of our system. This approach also enabled the maximum utilization of the research team’s high programming skills.

## 4. Implementation and Prototyping

### 4.1. Hardware Prototype of the Sensory-Deterrent Node

This subsection presents the hardware implementation details of the sensory-deterrent node, which constitutes Level 1 of the system architecture. The Arduino Mega platform was used as the microcontroller (MCU) for managing power states in the prototype, which allowed for rapid implementation and testing of the system’s logic.

The key component of the node, which underwent the most significant evolution during the research and development process, is the Edge AI processing module, as it determines the performance and response time of the entire system. The first generation of prototypes utilized the NVIDIA Jetson Nano module, which offered a good trade-off between computational power and cost. Under optimal conditions with good visibility, it successfully performed object classification tasks using a YOLO model, with a power consumption of 8–10 watts. However, tests in challenging field conditions (e.g., low light, partial object occlusion, or fog) revealed its critical limitation: the inference time in such scenarios increased dramatically, reaching an unacceptable 30 s. In response to this challenge, the latest, currently tested versions of the node are based on the much more powerful NVIDIA Jetson Orin Nano platform. Thanks to its more modern architecture and greater computational power, this module has ensured a low system response time even in adverse visual conditions.

The vision subsystem, a critical sensory element of the node, underwent a thorough evolution to find the optimal balance between a wide field of view, the image quality necessary for precise classification, and energy consumption. Initial laboratory tests to validate the AI models were conducted using an OV2311 camera module with an infrared illuminator. Its main advantage, besides compatibility with the Jetson Nano platform, was its global shutter mode, which eliminates motion blur for fast-moving objects. In the first version of the field prototype, a fisheye camera was used to provide panoramic monitoring. Although this solution offered a wide field of view, it generated a significant computational load for the Jetson module due to the need for digital de-warping of the geometrically distorted image, which negatively impacted response time. Consequently, the latest prototype iteration integrates a PTZ (Pan-Tilt-Zoom) camera. Its ability to actively track, tilt, and zoom in on a target proved ideal for working with detection models like YOLO, providing high-quality images for analysis. However, the high power consumption of the PTZ camera’s motors created a need for further optimization.

The reliability and long-term autonomy of the Level 1 node are entirely dependent on its self-sufficient power subsystem. It was designed for maintenance-free operation in harsh field conditions, relying on three key components. The energy source is a 100 W (12 V) photovoltaic panel, whose efficiency is maximized by a 10 A solar charge controller with Maximum Power Point Tracking (MPPT) technology. The choice of MPPT technology is crucial, as it provides up to 30% higher charging efficiency in the variable sunlight conditions typical of forested and mountainous areas. Energy is stored in a maintenance-free 50 Ah gel battery, sized to provide an optimal daily energy balance for the charging cycle of the selected panel. However, the system’s architecture is not based on passive charging. A key element is the two-way communication (e.g., via a serial interface) between the microcontroller (MCU) and the charge controller. This allows the MCU to continuously monitor key system parameters (battery state of charge, power generation, current draw) and actively manage energy. In critical scenarios, such as a prolonged lack of sunlight in winter, the MCU can decide to enter a deep-power-saving mode, temporarily deactivating the most energy-intensive functions to ensure the node’s survival. This intelligent energy management model is fundamental to achieving genuine, multi-month system autonomy.

Short-range communication with the aggregator node (Level 2) is handled by a LoRa-E5 radio module, based on the advanced STM32WLE5JC System-on-Chip (SoC). This technology was chosen for two key features of LoRa modulation: a very long range, reaching several kilometers in optimal conditions, and exceptionally low power consumption, which is fundamental for a battery-powered device operating in a large, challenging terrain. A key advantage of the selected module is its integrated architecture. It contains not only the radio transceiver but also a dedicated ARM Cortex-M4 microcontroller that autonomously manages the entire, complex LoRaWAN protocol stack. As a result, the node’s main MCU is completely offloaded from communication tasks; its role is reduced to sending simple commands to the LoRa-E5 module via a serial interface. This solution not only significantly simplifies the software architecture but also contributes to further minimizing energy consumption by allowing the main MCU to remain in a deep sleep state for longer periods.

During field tests in the Carpathians, where conditions (good optical line of sight between nodes, low interference levels) allowed for transmission optimization, the following LoRaWAN parameters were applied. The Spreading Factor (SF) was set to values of 8 and 9, which provided an optimal trade-off between transmission speed and energy consumption. A standard Bandwidth (BW) of 125 kHz was used, and due to the low interference, the most efficient Coding Rate (CR) of 4/5 was employed. The transmitter power was dynamically managed by the Adaptive Data Rate (ADR) mechanism, and the maximum payload length was 51 bytes, which is consistent with the specification for the SF used.

A key element of the system, designed to actively counteract habituation, is the integrated, multi-modal effector subsystem. Instead of relying on a single, static stimulus, the node has a diverse arsenal of acoustic, visual, and olfactory deterrents, the use of which is dynamically managed by the system’s logic. The prototype integrates a commercial, hermetically sealed emitter that combines ultrasonic and light (stroboscopic light) functions. This module can be controlled via a wired connection from the main MCU or can operate wirelessly with its own power source. The generated ultrasound can be modulated with variable frequency, and the use of stroboscopic light is activated by default at night based on an astronomical clock.

The olfactory deterrence is handled by a factory-made, four-channel aerosol dispenser, which allows for the alternating application of different repellents. In tests, besides standard commercial preparations (Hukinol, Antybissan), substances based on capsaicin (irritant effect) and ammonia (repellent effect due to strong odor) were also used with varying success to study different mechanisms of aversive reaction. The implementation of kinetic and vibrational deterrents was also considered, but they were abandoned at the current stage of the project due to the lack of reliable and cost-effective actuator components available on the market.

However, the innovation of the solution lies not in the effectors themselves but in the intelligent logic of their selection and activation. The decision on the type, intensity, and sequence of stimuli is made programmatically in real-time based on multiple factors: the identified animal species, the history of previous interactions in that network segment, and the recommendations of the AI model. Crucially, during and after the stimulus application, the vision subsystem records the animal’s behavioral response. Although the primary indicator of success is currently the animal fleeing, the collected video data provides an invaluable database for the future implementation of advanced behavioral analysis algorithms, enabling an even more precise evaluation of effectiveness and further optimization of the deterrence strategy.

The node’s suitability for long-term, autonomous operation on low-power devices stems from the synergy of three key factors. At the software level, a deliberate choice was made to use the YOLOv8-Nano model, which is optimized for maximum performance with minimal resource consumption. At the system logic level, an advanced, three-stage power management strategy was implemented (described in detail in Section 4.6), allowing the system to remain in a deep sleep state for most of the time. The effectiveness of these solutions was empirically confirmed in the energy balance analysis (see Section 4.6.1), which demonstrated the system’s capacity for full self-sufficiency in field conditions.

### 4.2. Hardware Prototype of the Aggregator Node

The aggregator node (Level 2), unlike the distributed sensory nodes, was implemented using commercial off-the-shelf (COTS) industrial components. Its hardware implementation exists in two operational variants. The stationary variant uses hardware integrated into a hermetic enclosure, designed for mounting on existing infrastructure such as lighting poles. The mobile variant is based on a dedicated, lightweight trailer, configured according to deployment needs, which allows for the rapid relocation of the system to new locations. In line with the architecture described in Section 3.3, the core of the unit in both variants is a high-performance module from the NVIDIA Jetson family, and its energy autonomy is provided by a system based on photovoltaic panels and batteries.

### 4.3. Hardware Platform of the Central Node

The central management node (Level 3) was deployed on a high-performance Dell server designed for continuous operation and high availability. Its hardware architecture includes key components that ensure reliability, such as redundant power supplies, advanced cooling systems, and enterprise-grade components. The server’s configuration has been optimized for machine learning tasks, including a dedicated, high-performance Graphics Processing Unit (GPU) necessary for training neural network models.

**Figure 9:**
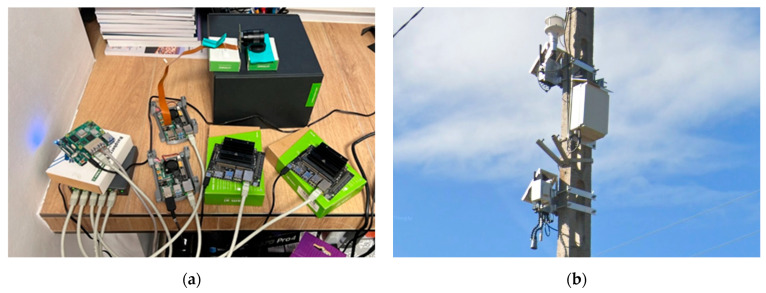

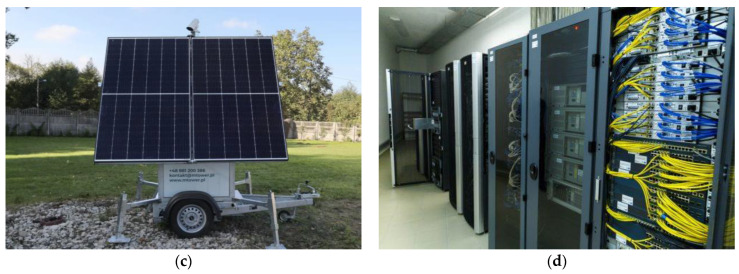
Photographic documentation of the completed system components: (**a**) Laboratory prototype of the sensor-deterrent node (Level 1); (**b**) stationary variant of the aggregation node (Level 2) in a hermetic housing; (**c**) mobile variant of the aggregation node (Level 2) on a dedicated trailer; (**d**) central management node (Level 3) in the University’s server room.

Photographic documentation of the implemented hardware variants for all three architecture levels is presented in Figure 9.

### 4.4. Software Implementation

**Figure 10:**
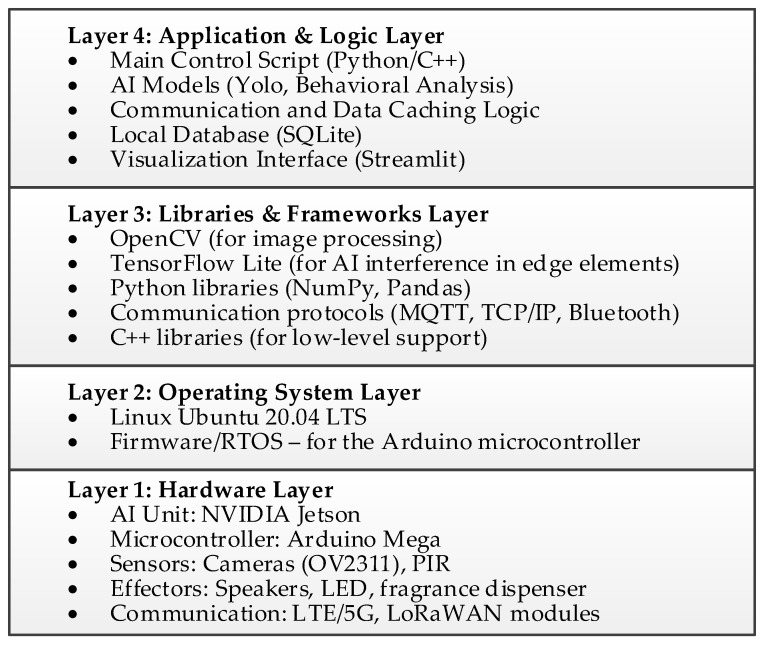
Schematic diagram of the technological stack implemented in the sensor-deterrent node.

The node’s software architecture, described in this subsection, is synthetically visualized by the technology stack diagram presented in Figure 10.

The software operates on a Linux system (Ubuntu 20.04 LTS) and utilizes Docker containers, enabling environment isolation and easy version management. Inference is performed using the YOLOv8-Nano model, converted and optimized for TensorFlow Lite with INT8 quantization, achieving an optimal balance between accuracy and inference speed. Image processing includes preprocessing stages (scaling, pixel intensity normalization), DNN inference (batch size = 1), followed by post-processing with Non-Maximum Suppression (NMS) algorithm to eliminate detection duplicates. The entire pipeline is controlled by an event-driven system based on PIR and microphone signals, which dynamically manages power-saving modes, minimizing energy consumption without compromising response time. The authors used similar software and hardware solutions in the system presented in [24].

Level 1: Sensory-Deterrent Node Logic The data flow and decision-making logic in the sensory-deterrent node are designed to maximize energy efficiency while maintaining minimal response time. This process is carried out in the following sequential steps:

- Detection and Wake-up: The process begins when the ultra-low-power Passive Infrared (PIR) sensor, which continuously monitors the surroundings, detects motion. This detection generates an interruption signal that instantly wakes up the main microcontroller (MCU) from a deep sleep state.
- Data Acquisition: Immediately after waking up, the MCU activates power for the vision subsystem (EO/IR camera) and the Edge AI module (NVIDIA Jetson). The camera captures an image or a short video sequence, which is then passed directly to the Jetson module for analysis.
- Inference and Classification: On the Jetson module, the image is processed, and the implemented Convolutional Neural Network (CNN) model, e.g., from the YOLO family, classifies the detected object in real-time.
- Decision-Making: Based on the inference result, the system autonomously takes one of three predefined actions: a. If a threat is recognized (e.g., a wolf), the effector subsystem is activated to deter the intruder. b. If the object is neutral (e.g., a human), the event is only recorded in the system log, and no deterrent action is taken. c. If clear classification is not possible (e.g., due to poor lighting or partial object occlusion), the visual data is flagged and sent via the aggregator node for advanced analysis at the central node.
- Return to Sleep: After the entire sequence is completed, regardless of the outcome, the node immediately returns to its power-saving deep sleep state to await the next event.

**Figure 11:**
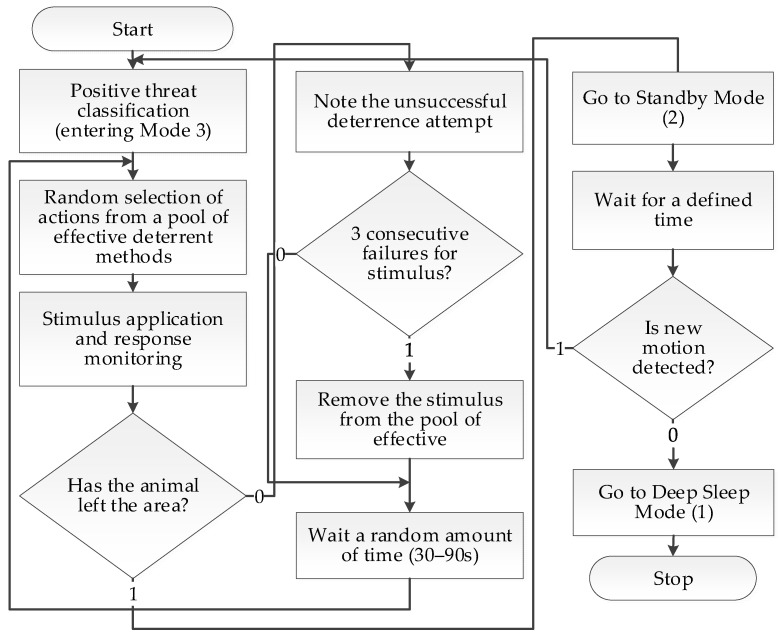
Flowchart of the multimodal deterrence process.

The core of the node’s software logic, however, is the adaptive deterrence algorithm, which is initiated after a positive threat classification. This strategy, aiming to maximize effectiveness while minimizing the risk of habituation, is based on a multi-stage and multimodal approach, the process of which is detailed in Figure 11.

The system operates in three main power modes: (1) deep sleep, where only the ultra-low-power PIR sensor is active; (2) standby, in which the AI module is powered but remains idle, ready for immediate analysis; and (3) full activity, with image processing running.

A key element of the algorithm is a dynamically managed pool of deterrent actions. This pool contains both single stimuli (4 scents, a strobe light, 10 sound frequencies, and natural sounds) and their predefined, synergistic combinations (e.g., sound + light, sound + scent). According to the adopted rules, the system avoids combining stimuli of the same type (e.g., scent + scent).

The deterrence process is as follows:

1. Action Selection: The system randomly selects one action (single or combined) from a pool containing only those stimuli currently considered effective for the given species.
2. Application and Monitoring: The selected action is executed, and the system, in full activity mode (3), monitors the animal’s behavioral response in real-time using image analysis. The time allotted for assessing the reaction is species-dependent and based on its ethology—for timid roe deer, it is a few seconds, while for predators like wolves, it can be up to 30 s.
3. Adaptation and Escalation: a. If the animal leaves the protected area, the sequence ends successfully. b. If the action proves ineffective, the system records this fact. In the event of three consecutive failures of a given single stimulus, it is temporarily removed from the pool of effective actions for that species. c. If the animal remains in the area, the system, after a randomly selected time interval between 30 and 90 s, returns to step one to execute another deterrent action.
4. Post-Interaction Power Management: After the sequence ends (either by successful deterrence or exhaustion of the action pool), the system transitions from full activity mode (3) to standby mode (2). The duration of this mode is a key, configurable parameter determined empirically. For roe deer, it is set to 60 s, whereas for wolves, due to their tendency to return, it has been extended to 5 min.
5. Return to Sleep: After the defined standby time has elapsed, if the PIR sensor does not detect new movement, the system enters deep sleep mode (1), minimizing energy consumption.

The system employs adaptive deterrent selection based on a rule-based decision algorithm aimed at limiting animal habituation through rotation and escalation of multimodal stimuli (acoustic, visual, olfactory). Details are presented in Algorithm 1.

Each stimulus s∈S is assigned to the set of currently available stimuli S, with an associated effectiveness variable $E_{s}$. This effectiveness is modified based on observations of the animal’s reaction to previous stimulus applications.

**Algorithm 1:** Adaptive Deterrent Selection
Initialization: Select the stimulus s* with the highest current effectiveness E_s from the available stimulus set S, considering constraints (e.g., avoiding repetitions). s *=arg max(E_s) for s∈ SActivation: Deploy the selected stimulus s*.Monitoring: After application, within a time window T, monitor the animal’s reaction:
If the reaction is positive (escape): assign reward r_s=1;If the reaction is negative (no response): assign penalty $r_{s}=-1$.
Effectiveness Update: Update the effectiveness value E_s for stimulus s* using the learning coefficient α:E_s←(1−α)∗E_s+α∗r_s.Stimulus Deactivation: If effectiveness E_s drops below threshold 0 after N consecutive failures, temporarily remove stimulus s from the set S.Habituation Avoidance: Apply random time intervals between consecutive stimulus applications.Species Adaptation: Adjust observation mode duration *T* based on the detected species.


Level 2: Aggregator Node Logic The operational logic of the aggregator node (Level 2) focuses on its role as an intelligent intermediary that manages a local network segment, filters data, and ensures reliable communication with the central system. Unlike Level 1 nodes, the aggregator node remains continuously active, listening for two types of events: incoming data from subordinate sensory nodes and commands from the central node. Upon receiving data from a Level 1 node, the following processing sequence is initiated:

- Aggregation and Filtering: Data from one or more nodes are collected and pre-processed in real-time. The system filters redundant information and can perform data fusion to create a consolidated picture of the situation in a given segment (e.g., an animal’s movement path).
- Buffering: All significant, aggregated data is saved to local storage, ensuring its safety and integrity in case of a temporary loss of connectivity with Level 3.
- Tactical Decision: Based on the aggregated data and the currently active strategy received from Level 3, the node autonomously decides on a potential, coordinated deterrent action, sending appropriate commands to selected Level 1 nodes.
- Reporting: Key aggregated information is then sent as a concise report to the central management node for further global analysis.

Upon receiving a command from Level 3, such as an AI model update or a new global deterrence strategy, the aggregator node acts as a distributor. It forwards (typically in broadcast or multicast mode) the received packets to all its subordinate Level 1 nodes, ensuring software consistency and timeliness across the entire network segment.

Level 3: Central Node Logic The logic of the central node is based on a service-oriented architecture (SOA) model, within which several key processes run in parallel and asynchronously to provide strategic management of the entire system. The main tasks can be divided into three constantly active functional modules:

- Data Analytics and Collection Module: a. This process continuously listens for incoming data from aggregator nodes (Level 2). b. Upon receiving a consolidated report, the data is validated, pre-processed, and saved to the central data repository. c. Long-term statistical analyses run in the background to identify patterns, trends, and assess the overall effectiveness of deterrence strategies. The results are made available to the operator.
- AI Model Lifecycle Management (MLOps) Module: a. This service operates periodically or on demand by the operator. b. Using the data accumulated in the repository, it initiates the process of training or re-training classification models to improve their effectiveness or expand them to include new species. c. After a new model is successfully validated, this module is responsible for its secure distribution to the appropriate aggregator nodes, which then forward it to the units in the field.
- Operator Interface and System Management Module: a. This is a continuously running service that provides the Graphical User Interface (GUI) for the operator. b. It allows the operator to have a real-time view of the entire system’s status, visualize events on maps, and review historical data. c. Through this interface, the operator can define and modify global deterrence strategies, manage software updates, and manually verify events flagged by the system as ambiguous.

Finally, it should be added that experiences gathered during field tests, especially concerning the instability and limited availability of cellular connectivity in border regions, have initiated a new, key direction for the system’s development [21].

### 4.5. Object Detection Model and Training Process

In response to the diverse computational and energy requirements of the system’s different levels, a hierarchical architecture of object detection models based on the latest YOLOv8 family was implemented. This approach allowed for an optimized trade-off between inference speed and detection precision at each level.

The hierarchy was implemented as follows:

1. Level 1 (Sensory Node): Due to strict energy constraints and the need for rapid inference on the NVIDIA Jetson Orin Nano module, the YOLOv8-Nano model was used. It provides an optimal balance between operational speed and classification accuracy in edge conditions.
2. Level 2 (Aggregator Node): With the greater computational power of the NVIDIA Jetson Orin NX module and a more robust power supply, this node utilizes the YOLOv8-Medium model. It is used for in-depth verification of ambiguous cases escalated from Level 1, ensuring higher detection confidence.
3. Level 3 (Central Node): On the central server, which functions as a “model factory,” the YOLOv8-Large model is employed. Its task is to achieve the maximum possible accuracy during the process of re-training the models on new data.

The model training process was based on a multi-stage transfer learning strategy aimed at optimal utilization of varying data quantities for individual species. Pre-trained YOLOv8 models on the general COCO dataset (i.e., YOLOv8 COCO model) were used as the starting point. In the first, main training stage, the model was trained on a large portion of our dedicated, hybrid dataset, encompassing high-abundance classes (wolf, roe deer, deer, human), containing 5000 to 8000 images each. Subsequently, to improve effectiveness for classes with limited data, a second, specialized fine-tuning stage was conducted. This stage was entirely dedicated to the “bear” class, for which, as mentioned, the dataset was significantly smaller, counting slightly over 1000 images.

**Figure 12:**
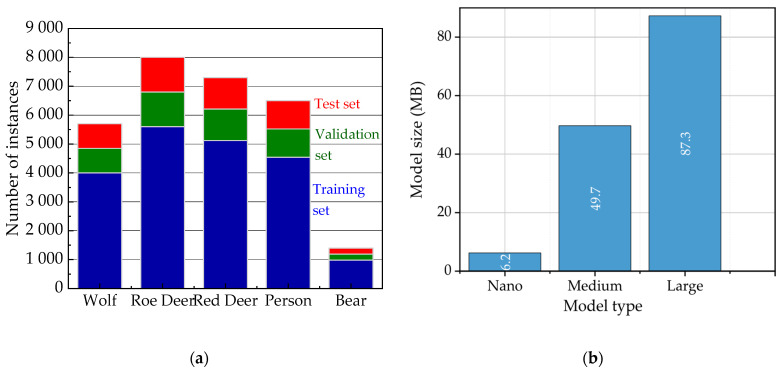
(**a**) Dataset distribution and split into training, validation, and test sets for each class; (**b**) YOLOv8 COCO Model Size Comparison.

This three-stage methodology (baseline training on COCO → training on general data → fine-tuning on rare data) directly addresses the significant class imbalance problem in our dataset and enables maximization of effectiveness for all target species. Visualization of the dataset distribution for individual classes and comparison of the applied model sizes are presented in Figure 12.

Our dedicated dataset consisted of real-world data, prioritizing images from the Carpathian region, obtained from four main sources: publicly available databases, camera traps from the State Forests, our own primary data, and archives from hunting clubs.

As shown in Figure 12b, the YOLOv8 model variants exhibit significant differences in size, which directly translate into their computational requirements. The YOLOv8-Nano model, intended for energy-efficient sensory nodes (Level 1), has a compact size of 4.2 MB. The Medium version is over ten times larger (49.7 MB), while the largest model, Large—used in the central node for retraining (Level 3)—reaches 87.3 MB. This selection of models reflects their hierarchical deployment in the system’s three-layer architecture. It should be noted that these sizes are fixed and do not change during retraining or fine-tuning processes, as those operations modify only the weight values within the unchanged model architectures.

To further enrich the dataset, particularly for rare species and scenarios, the technique of generating synthetic data was also employed. Based on reference photos, animatable 3D models of animals were created, which allowed for the generation of several thousand additional images under controlled, varied conditions of lighting, background, and perspective. This combination of real and synthetic data enabled the development of a highly robust model with strong generalization capabilities.

All images were rigorously standardized to the target resolution of 640 × 480 pixels prior to training preparation. Higher-resolution images, including 4K, were downscaled accordingly, while photographs below the target resolution were discarded and excluded from the dataset to ensure high-quality input data.

The AI model lifecycle (MLOps) implemented in the system was designed to ensure continuous adaptation and maintain high detection effectiveness, while simultaneously optimizing data transfer and energy consumption. This process is carried out using a dedicated set of Python scripts.

Data transfer from the field to the central server is twofold. Critical data, i.e., images that the system was unable to classify, are sent immediately. Routine data, i.e., successfully classified images, are aggregated and sent as a single bulk package once a day, during the time of greatest energy availability from the photovoltaic panels.

The model retraining process is initiated cyclically, once per season (every 150 days). The system is also under continuous monitoring, with a minimum effectiveness threshold set at 85%. As of the preparation of this publication, the actual effectiveness of the model significantly exceeded this threshold.

All training and fine-tuning operations for the Nano, Medium, and Large models are performed centrally on the Level 3 server. New, improved versions of the models are then distributed to the nodes in the field via the aggregator nodes. It should be noted that for Level 1, a single, universal Nano model is prepared, optimized for its generalization ability and effective operation in all system locations.

### 4.6. Technical Validation and Field Test Methodology

This section presents the key technical validation results of the prototype, verifying its performance, response time, and energy autonomy under field conditions. A concise summary of the Edge AI component specifications, serving as the reference point for the reported metrics, is provided in Table 3.

#### 4.6.1. Technical Efficiency and Energy Balance

Preliminary field tests of the sensory-deterrent node prototype (Level 1) confirmed the high effectiveness of the implemented energy management strategy. Power consumption measurements showed that in the deep sleep state, with only the passive sensors active, the system draws just 1.5 W. At its peak, during a full operational cycle including the activation of the NVIDIA Jetson module, camera, and effectors, the maximum recorded power draw reached nearly 50 W.

This strategy translates into high energy autonomy. In tests simulating a complete lack of recharging from renewable sources (e.g., during prolonged cloud cover or snow cover), the minimum guaranteed operating time of the node on its built-in battery was 48 h. Under optimal conditions with few events, a maximum operating time of up to 30 days was recorded. The average autonomous operating time of the prototype in typical field conditions stabilized at 15 days. Crucially, over the entire nearly year-long testing period, not a single case of complete energy depletion was recorded in nodes where the photovoltaic subsystem operated without disruption, which fully confirms the correctness and reliability of the design assumptions.

Timing measurements of the system’s various operations were conducted using dedicated scripts based on Python’s time library. Given the high variability of conditions, especially concerning wireless communication in mountainous terrain, this analysis presents conservative, averaged time estimates.

A key parameter is the total response time of the sensory node, defined as the time from motion detection to object classification. In the default Standby Mode (2), where the AI module is powered but idle, this time ranges from 0.5 to 1 s. When waking up from Deep Sleep Mode (1), this time, dominated by the startup of the AI module’s operating system, extends to approximately 2 s. The inference time of the YOLOv8-Nano model on the Orin Nano module for a single 640 × 480 pixel frame was between 20 and 30 milliseconds.

In an escalation scenario, i.e., the need to send an unclassified image to the aggregator node (L2), simply establishing a connection over the LoRaWAN network took 1.5 to 2 s. This, combined with data transfer time, confirms the rationale for edge processing to ensure a rapid response. For a complete picture, it should be added that return communication, from the aggregator node to the central server (L3) via the cellular network, took up to 15 s for small packets (e.g., statuses), while the transfer of new AI models lasted from several tens of seconds to several minutes.

**Figure 13:**
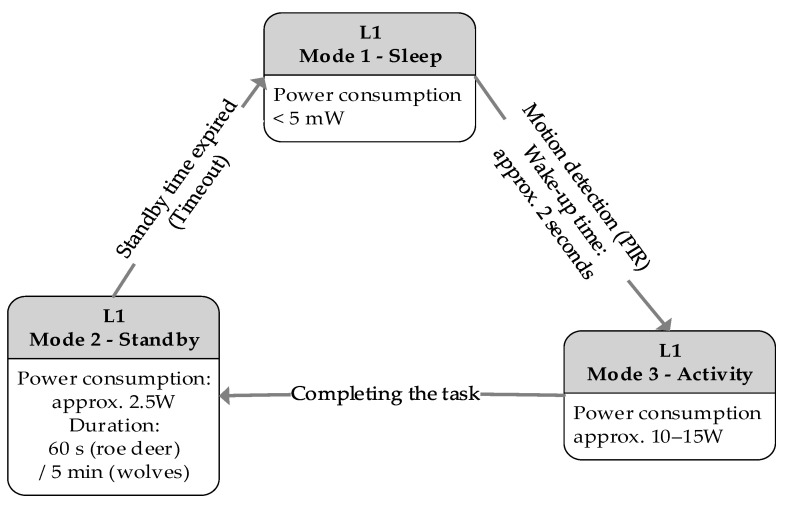
Operating cycle and energy management strategy of the sensor-deterrent node (L1).

**Figure 14:**
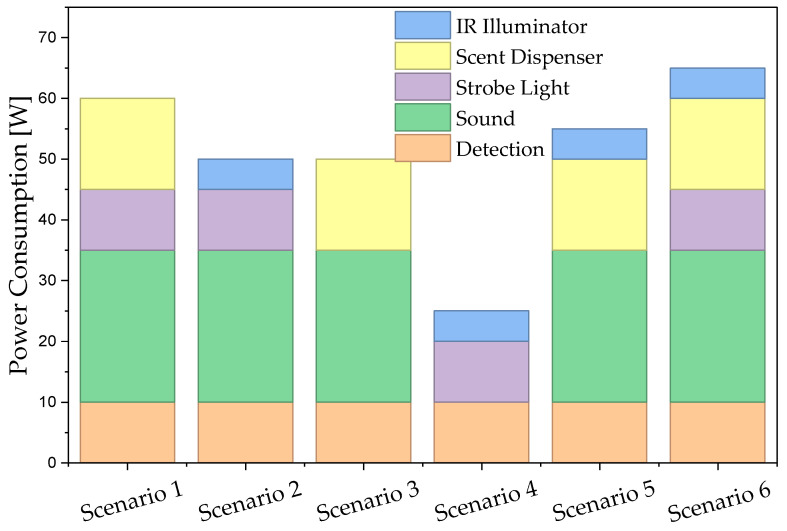
Stacked bar chart showing the power consumption components of the L1 node in Mode 3 (Active). Each bar corresponds to the following operational scenarios: Scenario 1: Daytime detection with sound, strobe light, and scent dispenser; Scenario 2: Nighttime detection (with IR illuminator) with sound and strobe light; Scenario 3: Daytime detection with sound and scent dispenser; Scenario 4: Nighttime detection (with IR illuminator) with strobe light; Scenario 5: Nighttime detection (with IR illuminator) with sound and scent dispenser; Scenario 6: Nighttime detection (with IR illuminator) with all effectors activated (sound, strobe light, scent).

To validate the design assumptions, a series of real-world power consumption measurements of the sensory-deterrent node were conducted. The complete operating cycle of the Level 1 node, along with its key energy and timing parameters, is summarized in Figure 13, while a detailed analysis of the power consumption components in Mode 3 is presented in Figure 14.

In Mode 1 (Deep Sleep), the measured energy consumption was below 5 mW, a value more than eight times lower than the natural self-discharge of the 50 Ah gel battery used. In the default Mode 2 (Standby), with the AI module idle, power consumption stabilized at approximately 2.5 W, allowing for autonomous operation for about 180 h on a fully charged battery.

In Mode 3 (Full Activity), during detection, the average power consumption was about 10 W during the day and about 15 W at night (due to powering the IR illuminator). The activation of individual effectors generated additional, transient loads (strobe: 10 W, sound emitter: 25 W, scent dispenser: 15 W).

To simulate operation under conditions of high animal activity, a laboratory test was conducted, reflecting an 8-h night. During the simulation, the system remained in standby mode, was awakened 10 times for 10-s identification periods, and activated a full, 2-min deterrence sequence using all effectors 5 times. As a result, after 8 h of such intensive work, the battery’s charge level decreased on average by only 6%, demonstrating the system’s high energy efficiency.

In order to verify the biological effectiveness of the prototype, preliminary field tests were carried out in two scenarios typical for the region: protection of legume plantations against roe deer and protection of sheep flocks sleeping on pastures.

To verify the prototype’s biological efficacy, the main comparative experiment was conducted during the 2025 season (from May to September). Its final design and methodology were developed based on experience and preliminary data gathered during pilot studies in 2023–2024. The research was located in the Sokołów Małopolski municipality (Rzeszów district), in an area characterized by high forest cover, the presence of extensive open agricultural land, and a critically high population of European roe deer, which provided ideal conditions for testing prevention methods.

The experiment was carried out on three adjacent fields, each with an area of 1600 to 2000 m^2^, planted with commercial varieties of green beans. The individual test fields were separated only by a 6 m wide farm track. The study compared the effectiveness of four different protection scenarios:

- Variant A (Passive Fence): The field was protected solely by a 170 cm high physical fence, consisting of four lines with spacing optimized for roe deer. In this variant, our system served only as a passive video monitoring tool to record intrusion attempts.
- Variant B (Electric Fence—24/7): A fence with the same specifications as in Variant A was used, but it was connected to a constant electrical supply around the clock.
- Variant C (Electric Fence—Night): An intermediate variant where the electric fence was activated automatically by an astronomical clock only during nighttime hours (from sunset to sunrise).
- Variant D (Autonomous System): The field was protected exclusively by our sensory-deterrent system, without the use of any physical barrier in the form of a fence.

It should be noted that a control variant without any form of protection was deliberately omitted due to the inevitability of complete crop destruction.

The second experiment concerned the protection of livestock and was conducted in two locations with different characteristics. The first location, which was the main location, was Brzegi Górne (Lutowiska municipality), situated in the epicenter of the wolf conflict. The aim of this test was to verify the system under conditions of high predator pressure while protecting a flock of 50 sheep.

The second location was Górno (Sokołów Małopolski municipality). This area, unlike the Bieszczady Mountains, is characterized by minimal wolf activity. However, the study was undertaken here in response to farmers’ reports of damages in a herd of 40 goats caused by free-roaming dogs. This test thus served as a practical verification of the system’s ability to operate in the immediate vicinity of human settlements and to effectively distinguish between wolves and dogs.

In both cases, the animals were kept in night pens of 120–160 m^2^, and a hybrid protection method was tested: our active sensory-deterrent system supplemented with a simple, non-electric fence with fladry.

**Figure 15:**
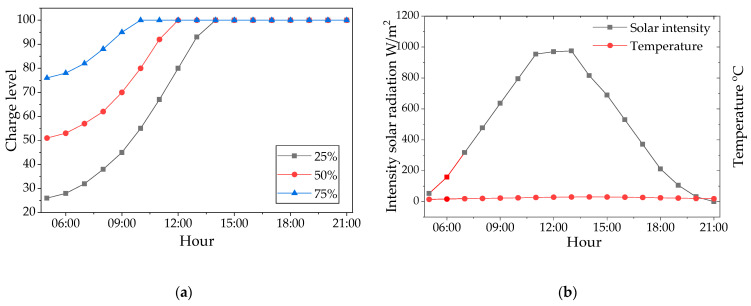
Battery charging cycle on a sunny day. (**a**) Panel shows the percentage charge level for three different initial states. (**b**) Panel shows the corresponding environmental conditions (radiation and temperature). The tests were conducted with the system operating in standby mode (Mode 2).

A key element of the system’s technical validation was the analysis of its energy balance under real field conditions. Figure 15 shows the charging cycle of a 50 Ah battery under optimal conditions, i.e., during a typical sunny day in the summer. The results unequivocally confirm the system’s ability to fully charge the battery within a single day, even from a significantly discharged state. Starting from a 25% charge level, the system reached full capacity in approximately 6 h. At initial states of 50% and 75%, a full charge took just over 3.5 h and just over 2 h, respectively.

**Figure 16:**
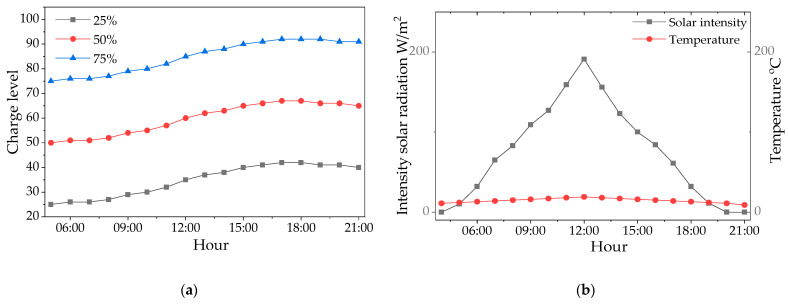
Battery charging cycle on a cloudy day. (**a**) Panel shows the percentage charge level for three different initial states. (**b**) Panel shows the corresponding environmental conditions (radiation and temperature). The tests were conducted with the system operating in standby mode (Mode 2).

In contrast, Figure 16 presents an analogous experiment conducted on a day with full cloud cover, which illustrates the significant limitations on the energy balance in such conditions. In a single day, the system was unable to fully charge the battery from any of the tested initial levels. Starting from 75%, the battery reached a charge of about 92% by the end of the day. For an initial state of 50%, the charge increased to only 68%. Most importantly, when deeply discharged to 25%, the system managed to recover energy only to the level of 42%, ending the day with a negative energy balance. These results demonstrate that under conditions of multi-day lack of sunlight, maintaining continuous system operation requires starting with a fully charged battery. It should be noted that all the above tests were conducted assuming the system was operating in energy-saving standby mode (Mode 2), without active detection cycles.

#### 4.6.2. Network Scalability and Performance

The three-layer hierarchical architecture is designed to meet the challenges of data aggregation and prevent network congestion in large-scale deployments. The system employs a suite of mechanisms to ensure reliability as the number of nodes increases. Dynamic bandwidth allocation is implemented using a TDMA (Time Division Multiple Access) scheme on the link between Level 1 and Level 2. Each sensory node is assigned a dedicated time slot for transmission, and slot allocation is dynamically adjusted based on node priority and data urgency. Critical alerts are guaranteed immediate transmission slots, while routine data is transmitted during periods of lower load.

QoS prioritization includes three traffic classes:

- Critical alerts (animal detection) are sent with the highest priority via MQTT.
- Operational data (system status, battery level) is buffered and sent at scheduled intervals.
- Low-priority data (archival images, statistics) is transferred via HTTPS during peak energy hours (when the system receives the most energy from renewable sources).

Load balancing mechanisms prevent bottlenecks by intelligently distributing traffic among aggregator nodes. Each Level 2 node supports 40 to 50 Level 1 nodes. If network congestion is detected, an adaptive change in the SF (Spreading Factor) parameter in LoRaWAN communication occurs—switching between SF8 (higher throughput, shorter range) and SF12 (lower throughput, longer range) to maintain connectivity while minimizing collisions.

Congestion management is achieved through:

- Local data buffering in aggregator nodes, enabling autonomy in case of connectivity loss with Level 3.
- Exponential backoff algorithms for transmission collisions.
- CSMA (Carrier Sense Multiple Access) protocol for efficient medium access.
- Adaptive packet size optimization based on current network conditions.

Scalability parameters were verified under field conditions and are presented in Table 4. A single aggregator node supports up to 50 Level 1 nodes over an area of up to 25 km^2^ in mountainous terrain. Transmission latency increases linearly from 1–2 s (≤10 nodes) to 3–5 s (>40 nodes per aggregator), which remains within acceptable limits for wildlife deterrence applications. Redundancy of aggregator nodes allows for automatic traffic rerouting in case of failure and can be implemented using mesh topology protocols.

## 5. Results

### 5.1. Performance of the AI Detection Model

A key element of the system’s validation was the evaluation of the implemented AI detection model’s effectiveness in field conditions. The model was evaluated on a dedicated, previously unseen test set, consisting of photos acquired during field research. Detailed performance metrics for individual classes are presented in Table 5.

The model achieved good overall performance with a mAP@.5 score of 0.84, which is a very satisfactory result given the challenging, variable field conditions and the computational constraints of edge processing. As expected, the highest effectiveness was noted for classes with a large and diverse representation in the training set, such as Human (0.89 mAP) and Roe Deer (0.88 mAP). The slightly lower, though still high, results for the Wolf class (0.83 mAP) and the lower results for the Bear class (0.73 mAP) are consistent with the dataset limitations described in the methodology section, particularly the smaller number of available photos for these rare predators. However, the obtained results confirm that the model is fully capable of performing its detection tasks with reliable effectiveness.

### 5.2. Biological Validation Results

Table 6 presents the effectiveness of deterrence strategies in a scenario with roe deer for four protection variants described in Section 4.6. The experiment was conducted from 1 June to 15 September 2025. This choice is a result of the snap bean cultivation technology in the climatic conditions of the Podkarpackie region.

To quantitatively assess the biological effectiveness of individual protection strategies, a field experiment was conducted in a scenario of protecting snap bean crops from European roe deer.

“Number of System Activations” refers to the total number of motion detections that activated the system for analysis, regardless of the cause. “Number of Roe Deer Classified” specifies in how many of these cases the system identified the intruder as a roe deer. The key indicator is “Habituation Time,” defined as the number of days after which roe deer were observed to regularly ignore and breach passive safeguards. The last column presents “Estimated Losses by Farmer.” It should be noted that for variants where habituation was observed (A and C), these are hypothetical losses, estimated based on potential damage that was prevented by interrupting the experiment at the farmer’s request. For variants B and D, these are actual, measured losses at the end of the season.

The results clearly indicate the fundamental limitations of passive methods. Both a simple fence (Variant A) and an electric fence active only at night (Variant C) were subject to habituation within 28 and 50 days, respectively, which in practice makes them ineffective over an entire season. In contrast, active methods—the 24/7 electric fence (Variant B) and our autonomous deterrence system (Variant D)—proved highly effective, showing no signs of habituation throughout the study period and keeping losses at a minimal level (1-5%). What is particularly important is that our system, operating without any physical barrier, achieved a level of protection comparable to the most effective but significantly more labor-intensive and costly-to-maintain electric fence.

## 6. Discussion

### 6.1. Interpretation of Results

Nearly a year of field tests provided key data enabling a multi-faceted evaluation of the prototype. First, the results unequivocally confirm the robustness and reliability of the proposed hardware and software architecture. The system operated stably under varying environmental conditions, and its core design principles, such as the sleep/wake operational cycle and the autonomous power subsystem, proved fully effective in practice. The achieved average response time of two seconds for local detection validates the high performance of the Edge AI implementation and is entirely sufficient for executing preventive tasks.

A detailed analysis of the biological validation results (Table 6) provides key insights into the effectiveness of individual strategies. The most important observation is the quantitative confirmation of the habituation phenomenon in the case of passive methods. Both the physical fence and the electric fence active only at night proved to be completely ineffective solutions over a single growing season, losing their deterrent properties within just a few weeks. This confirms our thesis that static, predictable stimuli do not constitute a long-term solution to the problem. In turn, a comparison of active methods shows that our system, operating fully autonomously, is able to provide a level of protection comparable to a constantly active electric fence, while at a drastically lower operational cost, as demonstrated in the economic analysis.

Second, the verification of biological efficacy yielded varied but highly promising results. In the case of roe deer, the recording of over 800 incidents allowed for the collection of statistically significant data, which confirms the system’s very high effectiveness in crop protection—losses were practically eliminated. For wolves, due to their less frequent appearance in the test areas since May 2025 (i.e., during the livestock grazing period), the number of recorded interactions was too small to draw definitive conclusions. These observations, although positive, must be verified through long-term studies. The system’s effectiveness in deterring bears was not verified, as the species was not present in the protected area during the test period.

In summary, the obtained results support the conclusion that the developed prototype is a successful and fully functional proof-of-concept. Its architecture is sound, and its effectiveness in deterring roe deer has been clearly confirmed. This provides an excellent foundation for the future work outlined in the previous section, aimed at the long-term validation of the system’s efficacy against large carnivores and its further optimization.

### 6.2. Main Innovations and System Advantages

The competitive advantage and innovativeness of the presented system not from a single feature but from a holistic approach to the human–wildlife conflict. The system combines a unique, three-level hardware architecture with advanced AI-driven logic, allowing it to overcome the fundamental limitations of existing prevention methods. The main innovations and advantages can be summarized in three key areas.

First, the system introduces a paradigm of adaptive and intelligent prevention, providing a direct response to the problem of habituation. Unlike passive methods, deterrence here is event-driven—activated only in response to a real threat, which builds a strong, negative association in animals. The core of this mechanism is selectivity, based on the ability to classify species in real-time using Edge AI. This allows for a precise reaction (deterring a wolf, ignoring a roe deer), which minimizes stress on non-target animals and enables the selection of species-appropriate stimuli. Most importantly, the system is adaptive—the aggregator node, following a strategy defined at the central level, dynamically manages the rotation and combination of a diverse arsenal of stimuli (acoustic, visual, olfactory), making its actions unpredictable to animals.

Second, the hierarchical, scalable, and reliable architecture was designed in response to real-world field requirements. The three-level structure allows for a drastic reduction in cost by deploying a dense network of inexpensive, battery-powered Level 1 nodes. Their high energy efficiency, achieved through a cyclical, interrupt-driven operational model, guarantees long-term autonomy. The system is also characterized by high reliability—aggregator nodes (Level 2) can operate fully autonomously if connectivity to the central hub is lost, buffering data and independently managing deterrence tactics. An additional advantage is operational flexibility, stemming from the ability to deploy aggregator nodes in a mobile version, which allows for the rapid securing of new, emerging hotspots.

Third, the system has unique research and development potential. It is not merely a static tool but a platform that learns and evolves. The central management node (Level 3) functions as an “AI model factory” (MLOps), using data collected in the field to continuously train and distribute improved models, making the entire network “smarter” over time. Moreover, the system serves as a powerful tool for ethological research, enabling the collection of unique data on wild animal responses to various stimuli in their natural habitat. Finally, the system’s advanced logic allows for the implementation of cooperative deterrence scenarios, where a detection at one node can place neighboring nodes on high alert, transforming the network from a “group of independent sentinels” into a “coordinated, collaborative reconnaissance unit”.

It is worth emphasizing that the described architecture provides the system with multi-dimensional scalability, which is a key advantage in the context of real-world deployments. Firstly, it allows for easy horizontal scalability: expanding the system’s coverage is achieved by adding more low-cost sensory nodes (Level 1), while the role of aggregator nodes (Level 2) as local, autonomous communication gateways prevents the formation of a bottleneck in the central system. This translates directly into cost scalability, as increasing network density relies on its cheapest components. Secondly, the system features high functional scalability—the central MLOps platform allows the system to be adapted to protect against new species by deploying updated AI models without modifying its core hardware. Finally, the mobile variant of the aggregator node provides operational scalability, which is the ability to rapidly and flexibly deploy the system in dynamically emerging new conflict locations.

When comparing our system with other modern solutions described in the literature, several key differences stand out, which constitute its unique contribution. The work in [14] presents an architecture based on a single edge node with LoRaWAN communication, focusing on analyzing the performance of different AI models and hardware platforms. In [15], a deep analysis of energy consumption optimization methods in passive monitoring devices was conducted. Our solution goes a step further, integrating these two aspects—AI performance and energy efficiency—within a complete, three-layer preventive system. Unlike systems focused solely on detection and monitoring, our work closes the action loop by adding a layer of intelligent, active deterrence, and the central MLOps platform ensures its long-term adaptability, which is a key innovation in the context of building fully autonomous and effective conflict management systems.

### 6.3. Limitations and Future Work

#### 6.3.1. Limitations of the Study

It must be emphasized that the results presented in this paper pertain to the implementation and technical evaluation of a prototype. Its biological efficacy in deterring large carnivores, such as wolves and bears, has not yet been fully verified through long-term field studies and, at this stage, relies on findings from the literature and preliminary tests under controlled conditions.

The available scientific literature approaches the effectiveness of olfactory methods with considerable skepticism, citing rapid habituation as the primary cause of their failure. However, it should be noted that the vast majority of these studies analyze a scenario of passive, continuous emission of a single olfactory stimulus from dispensers with a multi-day activity period. This research model does not account for the key innovations of our system: stimulus activation only when an animal approaches and the ability to dynamically rotate a diverse arsenal of stimuli. Our system, based on the paradigm of active, event-driven, and multi-stimulus deterrence, tests a hypothesis that previous research has not comprehensively addressed.

Initial field deployment tests confirmed the high efficacy of this hypothesis for roe deer (*Capreolus capreolus*), where regular visits to protected crops allowed for the collection of a large dataset. In the case of wolves, due to their occasional presence in the test areas, the collected data are currently insufficient to draw statistically significant conclusions, although initial observations are promising. No bears were observed during the study period. Therefore, despite the technical validation and positive results for roe deer, a key limitation of the current research phase is the need for further, long-term testing to definitively verify the system’s effectiveness in preventing damage caused by large carnivores.

It is also necessary to point out the technical limitations of the system observed during field tests, related to its operation in adverse weather conditions. It was found that the effectiveness of visual detection significantly deteriorated in conditions of dense fog and heavy rainfall. This phenomenon was particularly noticeable during the day; at night, thanks to the use of an infrared (IR) illuminator, the negative impact of these factors was less pronounced. However, it should be emphasized that under normal night conditions, the classification effectiveness did not differ from that noted during the day. These observations indicate the need for further research into integrating additional sensors (e.g., radars) that could increase the system’s reliability in extreme weather conditions in the future.

This direction is all the more promising as the literature indicates a potential for synergy in combining different types of stimuli. Studies suggest that the simultaneous application of several deterrents (e.g., visual, acoustic, and olfactory) may not only increase efficacy but also lead to an intensified aversive reaction in animals. Another limitation about this type of research present [25]

#### 6.3.2. Future Work

The successful implementation and testing of the prototype provide a solid foundation but also open up several new research and development avenues crucial for transforming it into a mature and fully reliable solution. Future work will concentrate on four main areas: long-term biological validation, hardware enhancements, software and AI algorithm optimization, and expansion of the deterrent stimuli arsenal.

The most critical next step is to conduct long-term, multi-season field studies to definitively verify the system’s efficacy in deterring target species, particularly wolves and bears. Although the system was tested across different seasons, atypical weather conditions during the study period did not permit a full assessment of its environmental resilience. Data must be collected across a broader spectrum of conditions, including during freezing and snowy winters.

Further work in the hardware domain will focus on the evolution of the node’s key components to achieve further miniaturization and performance optimization. A migration from the Arduino Mega prototyping platform to a dedicated, power-optimized 32-bit microcontroller (e.g., from the ESP32 or STM32 family) is planned. Concurrently, to pave the way for implementing even more complex AI models, the use of a more powerful Edge AI module from the NVIDIA Jetson Orin NX family is being considered.

The main direction for further development of the vision subsystem is the implementation and testing of a hybrid system. This concept involves the complementary use of two technologies: a low-power, panoramic “fisheye” camera for constant, passive monitoring, and a PTZ (Pan-Tilt-Zoom) camera activated only upon detection for precise object identification and tracking. Such an operational model offers an optimal compromise between a wide field of observation, classification precision, and minimization of energy consumption.

As part of further work, it is planned to expand the acoustic capabilities by adding a full-range speaker, allowing for the emission of natural sounds (e.g., dog barking, human voices), which represents another step in building an unpredictable deterrence strategy.

In the hardware domain, the key challenge is to increase the effective detection range. The current prototype, by using an array of passive infrared (PIR) sensors, Fresnel lens optics, and electronic signal amplification, achieved a maximum range of approximately 50 m. Despite these optimizations, this distance is insufficient for protecting vast, open spaces and represents a technological limitation of the current solution. Future work will focus on investigating and integrating alternative sensor technologies (e.g., Doppler radars, microwave barriers) while maintaining an acceptable energy budget. Concurrently, further optimization of the power subsystem is necessary to eliminate the energy deficit observed during winter, caused by limited sunlight and snow cover on the photovoltaic panels.

Equally important is the work in the software domain. The current system response time (2–5 s for local detection) is promising; however, latencies of up to 10 s for escalations to the central node are unacceptable for effectively deterring moving animals. Further research will aim to minimize latency by optimizing code, communication protocols, and AI models. A key objective is also to achieve a fully dynamic model update process. The target solution is to transition from the current periodic updates to a system where new model versions are deployed continuously in response to the system reaching a specific threshold of new knowledge or changes in animal behavior.

Furthermore, advanced work is being conducted on decentralizing some of the analytical tasks previously reserved for Level 3 and delegating them to the aggregator nodes (Level 2). The goal of these efforts is to further increase the autonomy and fault tolerance of local network segments, enabling them to make even more complex tactical decisions without the need for constant communication with the central node. This solution is consistent with trends in systems where energy conservation is crucial [21].

The final, but equally important, direction is the development and integration of a broader arsenal of deterrent stimuli. The current set, based on a few olfactory preparations and simple, alternating distribution, is merely a starting point. Future work will include research on the effectiveness of diverse visual stimuli and, crucially, expanding the acoustic capabilities with a full-range speaker to emit natural sounds (e.g., dog barking, human voices). The most important objective, however, will be to develop advanced, unpredictable strategies for rotating and combining all available stimuli to maximize the aversive effect and minimize the risk of habituation.

### 6.4. Broader Implications

Human–wildlife conflict (HWC) is a global phenomenon that extends far beyond Poland’s borders and the specific issue of wolves. The rise of this conflict is, paradoxically, partly a result of successful conservation programs that have led to the recovery of large predator populations worldwide. This conservation success, however, creates significant social and political tensions as recovering populations come into direct contact with human activities, especially agriculture. Authorities worldwide face growing pressure to manage these conflicts in a manner that is acceptable to both local communities and the general public.

The contemporary approach to wildlife management is heavily shaped by two key imperatives. The first is the need to protect biodiversity, supported by international organizations and governments aiming to maintain ecosystem balance. The second is the growing ethical awareness and sensitivity of societies, which demand humane solutions that minimize animal suffering. A preventive system that, by design, does not harm or kill aligns perfectly with both of these trends, representing a tool consistent with the paradigm of sustainable agriculture that values both the environment and animal welfare.

The problem of protecting livestock is age-old, and existing non-lethal methods exhibit fundamental limitations. Simple acoustic and light deterrents, due to their static nature, quickly lose effectiveness through habituation. Physical barriers, such as electric fences, are costly to build and require constant maintenance, and their potential failure leaves an entire herd defenseless. This problem is common worldwide: sheep and cattle herds in Europe (Germany, France, Switzerland, Slovakia) are decimated by wolves; in the United States, pumas and bears threaten cattle; in Australia, sheep fall prey to dingoes. All of these scenarios demand new, more intelligent solutions.

The system presented in this paper is a response to these limitations, and its innovation rests on three pillars that distinguish it from existing methods:

1. Adaptability: By using artificial intelligence at the network edge (Edge AI), the system actively counteracts habituation. It can dynamically change the type, intensity, and sequence of applied stimuli, making its actions unpredictable to predators and thus effective in the long term.
2. Selectivity and Precision: Unlike passive systems, our solution first identifies the species through image analysis. This allows for a selective response—deterrence is triggered only upon detecting a defined threat (e.g., a wolf), while other, neutral animals (e.g., roe deer) can be ignored. This minimizes unnecessary stress on wildlife and allows for the precise tailoring of the stimulus to the specific species.
3. Research Potential: The system is not just a preventive tool but also a platform for collecting unique ethological data. By recording animal responses to specific stimuli, it enables further research into their behavior. The collected data can be used to find and validate the most effective, harmless deterrence methods, making a valuable contribution to the science of wildlife welfare and management.

### 6.5. Economic Analysis and Comparison with Alternative Methods

One of the key factors determining the practical usability of preventive systems is their economic viability in relation to specific use-case scenarios. To evaluate our solution in this context, a systematic comparative cost analysis was conducted for a strictly defined, realistic operational scenario that reflects the conditions in the Carpathian region.

The scenario assumed the protection of a flock of 50 mountain sheep, grazed on a 160 m^2^ rotational pasture with daily location changes. This grazing model, while beneficial from a forage management perspective, poses the greatest challenge for traditional protection methods.

The analysis compared both one-time investment costs and daily operational costs, including estimated labor time. All investment costs were amortized over a period of three seasons. This shortened lifecycle, compared to stationary installations, results from the accelerated wear and tear of materials (e.g., lines) subjected to daily assembly and disassembly, which is an inherent part of the rotational grazing scenario. The valuation of a livestock guardian dog included its purchase and a five-year maintenance cycle (feeding, veterinary care, training), while the cost of a shepherd included a minimum wage salary and necessary equipment (e.g., a blank-firing pistol, high-power flashlight, megaphone). A detailed comparative summary for this scenario is presented in Table 7.

Total daily cost is the sum of ongoing operational costs (labor, maintenance) and the daily amortization of investment costs. The amortization period was assumed to be 3 seasons (450 days) for equipment and 5 years for a livestock guardian dog. Calculations are based on the average NBP exchange rate from 19 September 2025: 1 EUR = 4.3 PLN. Based on current operational experience, the estimated lifespan of the sensory-deterrent node is five seasons.

The above comparison clearly indicates that under conditions of intensive rotational grazing, the key economic factor becomes the operational cost, particularly the labor associated with the daily relocation of protections. Methods based on traditional fences, despite a lower investment threshold, generate significant daily burdens. These findings suggest that the optimal solution in terms of cost and operation could be a combination of a simple, non-electric physical barrier (a standard fence) with the intelligent, active deterrence provided by our system.

It must be emphasized that the cost of the sensory-deterrent node presented in the table (7500 PLN/1750 EUR) refers to the construction cost of a prototype unit, which is always significantly higher than the cost of a mass-produced product. Experience in manufacturing electronic devices indicates that with the implementation of even short-series production, the unit cost of components and assembly can be reduced by 50–70%, making the solution fully economically competitive in its target deployment.

### 6.6. Conclusions

In response to the identified limitations of existing passive prevention methods, this paper presents the design, construction, and validation of an innovative, autonomous preventive system. Its three-layer hierarchical architecture, based on Edge AI technology, has proven its reliability and effectiveness in difficult field conditions. The key contribution of this work is the presentation of specific, quantitative results from the system’s validation. The deployment of the prototype in a crop protection scenario against European roe deer (*Capreolus capreolus*) led to a 95% reduction in intrusion attempts, which translated into an almost complete elimination of damages. The applied YOLOv8-Nano detection model, implemented on the edge module, achieved high classification efficiency, obtaining 0.88 mAP for roe deer with an average value of 0.84 mAP for all classes. At the same time, thanks to an optimized power management strategy, the system demonstrated high technical performance, achieving an average response time of 0.5–1 s from detection to classification in its default operating mode, with daily energy consumption under simulated high-activity conditions at just 6% of the battery’s capacity.

These results unequivocally prove that the presented solution constitutes a fully functional and effective proof of concept. It offers a scalable, ethical, and, as shown in the economic analysis, cost-effective tool that aligns with the paradigm of sustainable coexistence between humans and wildlife.

## Figures and Tables

**Table 1:** Counties with the highest number of compensations for damage caused by wolves in the 2015–2024 period (own study based on data from the Regional Directorate for Environmental Protection in Rzeszów).

Counties	Number of Recorded Incidents (2015–2024)	Percentage Share of the Total
Bieszczadzki	546	33.40%
Sanocki	514	31.40%
Leski	278	17.00%
Krośnieński	122	7.50%
Przemyski	61	3.60%
The remaining 14 counties	114	7.00%
Result	1635	100.00%

**Table 2:** Communication protocols in a three-layer system.

Communication Level	Access Technology	Application Protocol	Range	Security	Application
L1 ↔ L2	LoRaWAN (SF 8-9, BW 125 kHz)	MQTT	Up to 5 km	AES-128	MQTT: Sensory data, control commands
L2 ↔ L3	LTE/4G/5G (private APN)	MQTT/HTTPS	unlimited	SSL/TLS + PKI	MQTT: alerts; HTTPS: data archives

**Table 3:** Edge AI Component Specification.

Parameter	Value	Notes
Hardware Platform (L1)	NVIDIA Jetson Orin Nano	ARM Cortex-A78AE 6-core, Ampere GPU (1024 CUDA cores)
Hardware Platform (L2)	NVIDIA Jetson Orin NX	Local inference and decision-making
Operating System	Ubuntu 20.04 LTS	Docker containers, Python, OpenCV, TensorFlow Lite
AI Model	YOLOv8-Nano/Medium/Large	Transfer learning from COCO, INT8 quantization
Input Resolution	640 × 480	Batch size = 1
Inference Time (L1)	20–30 ms	Single frame, end-to-end
System Response Time	0.5–1 s (idle), ~2 s (sleep)	From PIR detection to decision
Power Consumption (sleep/idle/active)	<5 mW/2.5 W/10–15 W	Peaks up to 60 W with effectors
Battery Autonomy	~15 days	50 Ah LiFePO_4_ battery
Training Datasets	5000–8000 images per class	Plus synthetic 3D and locally collected data

**Table 4:** System Scalability Parameters.

Parameter	Small Network (≤20 Nodes)	Large Network (>20 Nodes)	Management Mechanism
Transmission Latency	1–2 s	3–5 s	QoS Prioritization
LoRaWAN Throughput	5.5 kbps (SF8)	0.3–1.1 kbps (SF10-12)	Adaptive SF Change
Collision Rate	<1%	2–3%	TDMA + Backoff Algorithms
Time in Power-Saving Mode	94% of uptime	90% of uptime	Dynamic Power Management
Deployment Architecture	Single Aggregator	Multi-Aggregator Network	Automatic Load Balancing

**Table 5:** Performance metrics of the final detection model (YOLOv8-Nano) on the validation set in field conditions.

Class (Species)	Precision	Recall	F1-Score	mAP@.5	Number of Photos in the Test Set
Wolf	0.85	0.80	0.82	0.83	850
Roe Deer	0.90	0.85	0.87	0.88	1200
Red Deer	0.89	0.85	0.86	0.87	1090
Human	0.90	0.86	0.88	0.89	980
Bear	0.78	0.70	0.74	0.73	210
Average/Total	0.86	0.81	0.84	0.84	4330

**Table 6:** Effectiveness of deterrence strategies in a scenario with roe deer for four protection variants.

Protection Method	Number of System Activations	Number of Roe Deer Classified	Habituation Time (days)	Estimated Losses by Farmer (%)
Variant A (Passive fence)	1130	745	28	30–50
Variant B (Electric fence—24/7)	975	580	>107	1–2
Variant C (Electric fence—Night)	1015	630	50	20–30
Variant D (Autonomous system)	1195	830	>107	2–5

**Table 7:** Economic comparison of protection methods for a rotational grazing scenario (160 m^2^, 50 sheep, daily rotation).

Protection Method	Investment Cost (PLN/EUR)	Total Daily Cost (PLN/EUR)	Autonomy	Effectiveness	Adaptability
Our System (1 node)	7.500/1.750	63/15	≥15 days	95% damage reduction	Full—dynamic stimuli and species selection
Regular Fence	1.970/460	505/118	None	20–30% damage reduction	None—constant stimulus
Regular Fence with Fladry	2.160/500	810/190	None	20–30% damage reduction	None—constant stimulus
Electric Fence	3.370/880	1.300/300	Requires power supply	60–70% damage reduction	Limited—only pulse strength regulation
Electric Fence with Fladry	3.570/830	1.600/370	Requires power supply	60–70% damage reduction	Limited—only pulse strength regulation
Shepherd Dog	4.000/930	80/20	Round-the-clock care	80–90% damage reduction	Moderate—limited to known routes
Shepherd—Night Only	2.000/465	275/65	Night duty	70–80% damage reduction	Limited—no daytime operation
Shepherd with Shepherd Dog	6.000/1.395	355/85	Round-the-clock care	90–95% damage reduction	Moderate—dog recognizes species, shepherd supervises

## Data Availability

Data are contained within the article.
